# Supplementary material for: Shrinkage improves estimation of microbial associations under different normalization methods
Source: NAR Genom Bioinform. 2020 Dec 17;2(4):lqaa100. doi: 10.1093/nargab/lqaa100 (PMC7745771; doi:10.1093/nargab/lqaa100)
Supplement: lqaa100_Supplemental_File [file lqaa100_supplemental_file.pdf]

1 Supplementary Figures

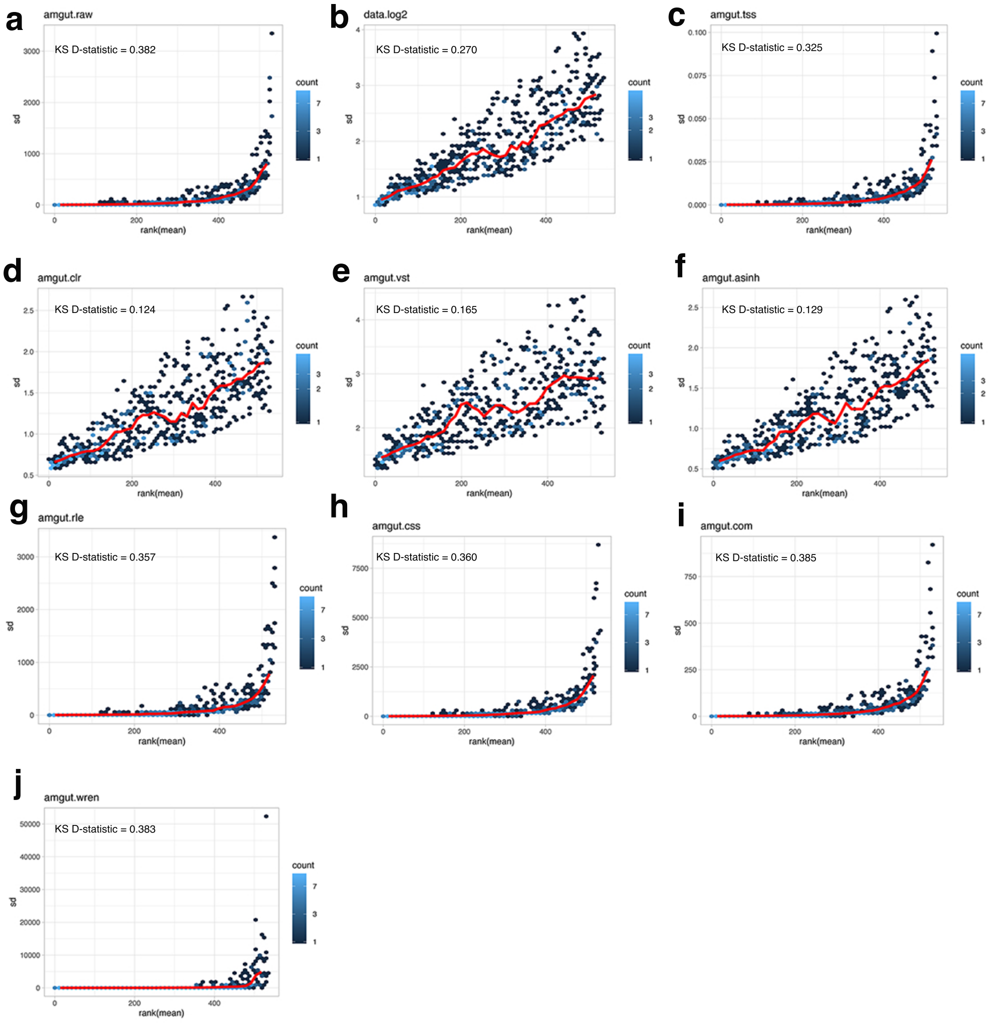

Figure S1: Standard deviation per-OTU, taken across all samples of transformed data plotted against the rank of the average count. These plots display the effect of normalization on the mean-variance relationship. The Kolmogorov-Smirnov normality test D-statistic represents the average across 531 normality tests for each OTU. A higher D-statistic value indicates a greater distance between the cumulative density function of the distribution of transformed data from that of a normal distribution. See Supplementary Methods for details.

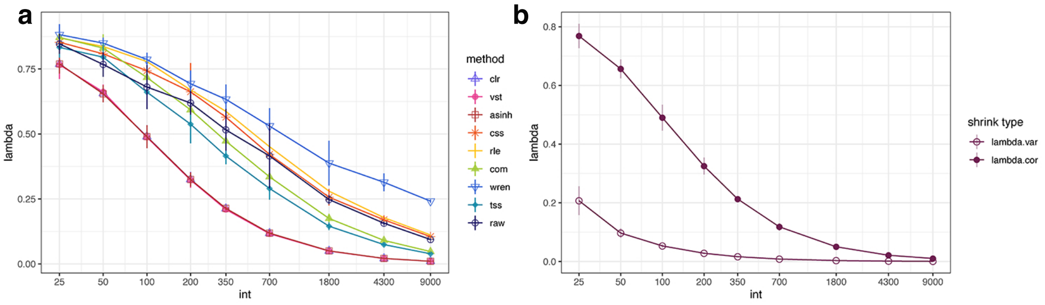

Figure S2: Lambda values for shrinkage estimation. a) Lambda values selected for shrinkage estimation of correlation ($\widehat{\lambda}_1^*$). b) Lambda of correlation ($\widehat{\lambda}_1^*$) and Lambda of variance shrinkage ($\widehat{\lambda}_2^*$) used jointly to estimate shrinkage of covariance for rhoshrink)

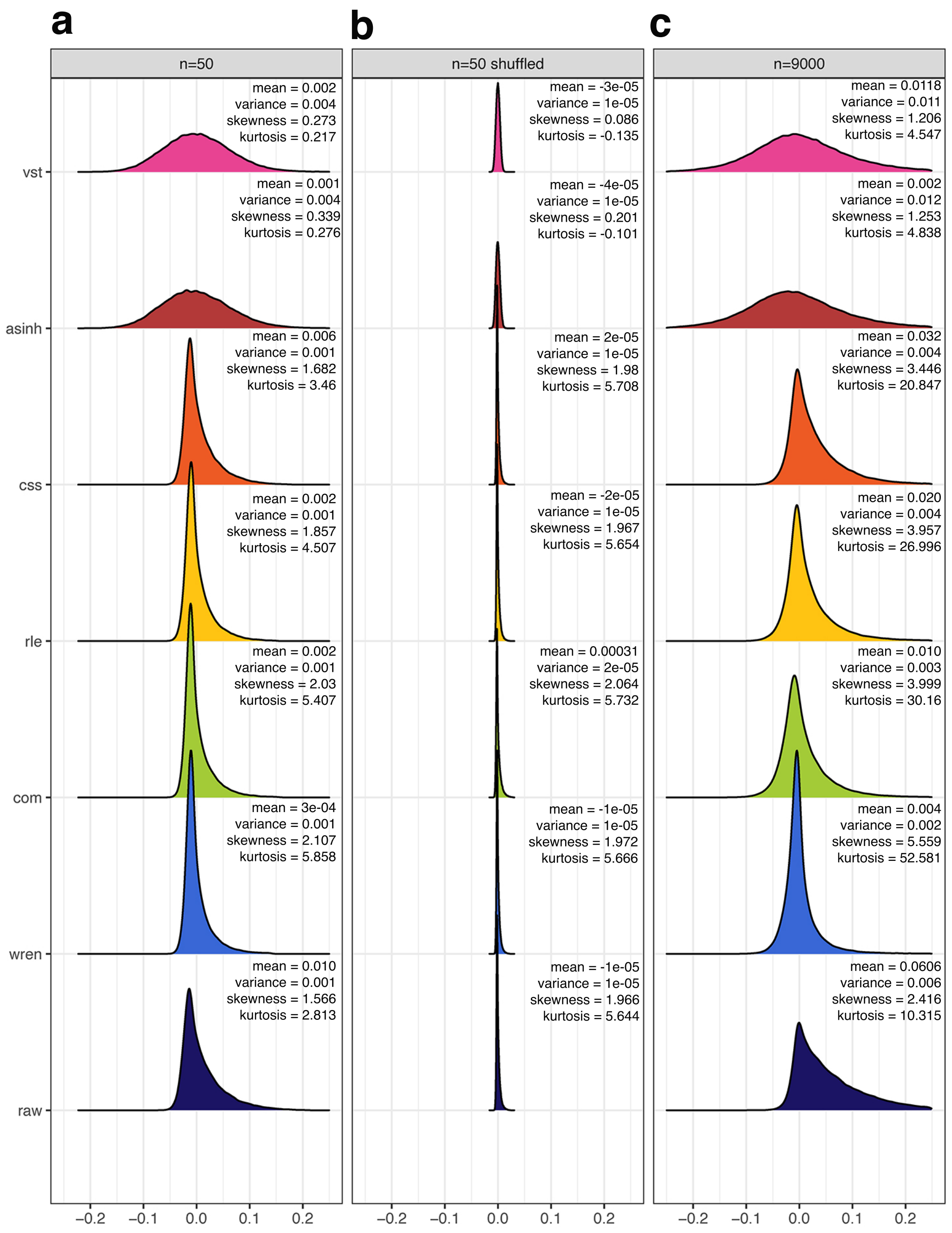

Figure S3: Density of association values after transformation and shrinkage. Each plot is a single random subsample of four representative methods at a) 50 samples, b) 50 samples with shuffled data and c) 9000 samples. Mean, variance, skewness and kurtosis are shown for each distribution.

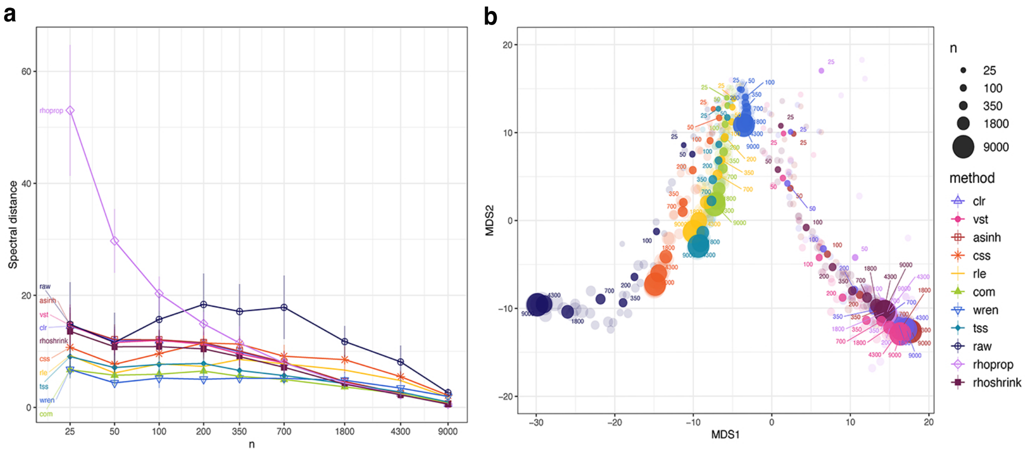

Figure S4: Spectral distance between estimates of association a) Spectral distance between sub-samples of different sizes. Lines represent mean and error lines represent standard deviation from the mean. Lines represent normalized matrices where correlation/proportionality estimation with shrinkage was performed. b) Multidimensional scaling representation of Spectral distance between correlation structures of varying size estimated from different normalization methods. The most opaque points represent the mean of 5 subsamples of the same size. (color scheme as in A)

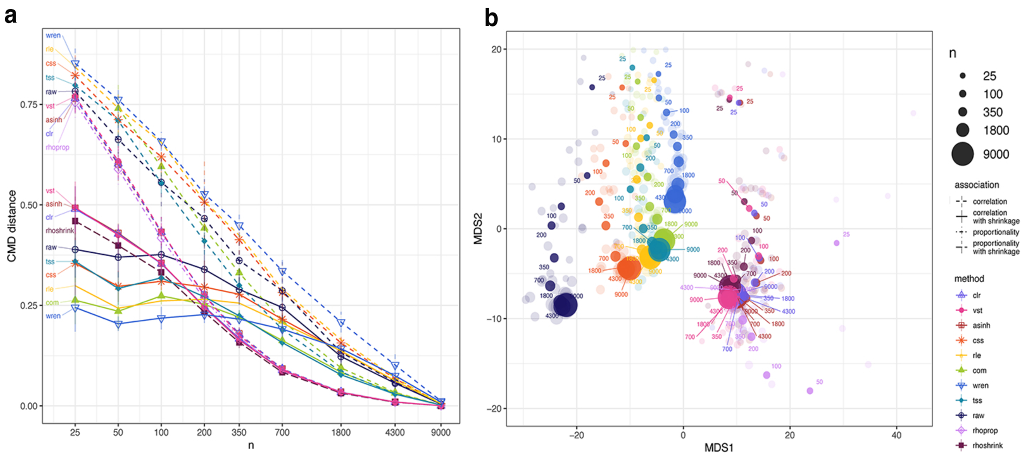

Figure S5: CMD distance between estimates of association a) CMD distance between sub-samples of different sizes. Lines represent mean and error lines represent standard deviation from the mean. Dashed lines represent normalized matrices after Pearson correlation. The solid lines represent normalized matrices where correlation/proportionality estimation with shrinkage was performed. The dot-dash line represents rho, a proportionality metric. b) Multidimensional scaling representation of CMD distance between correlation structures of varying size estimated from different normalization methods. The most opaque points represent the mean of 5 subsamples of the same size. (color scheme as in A)

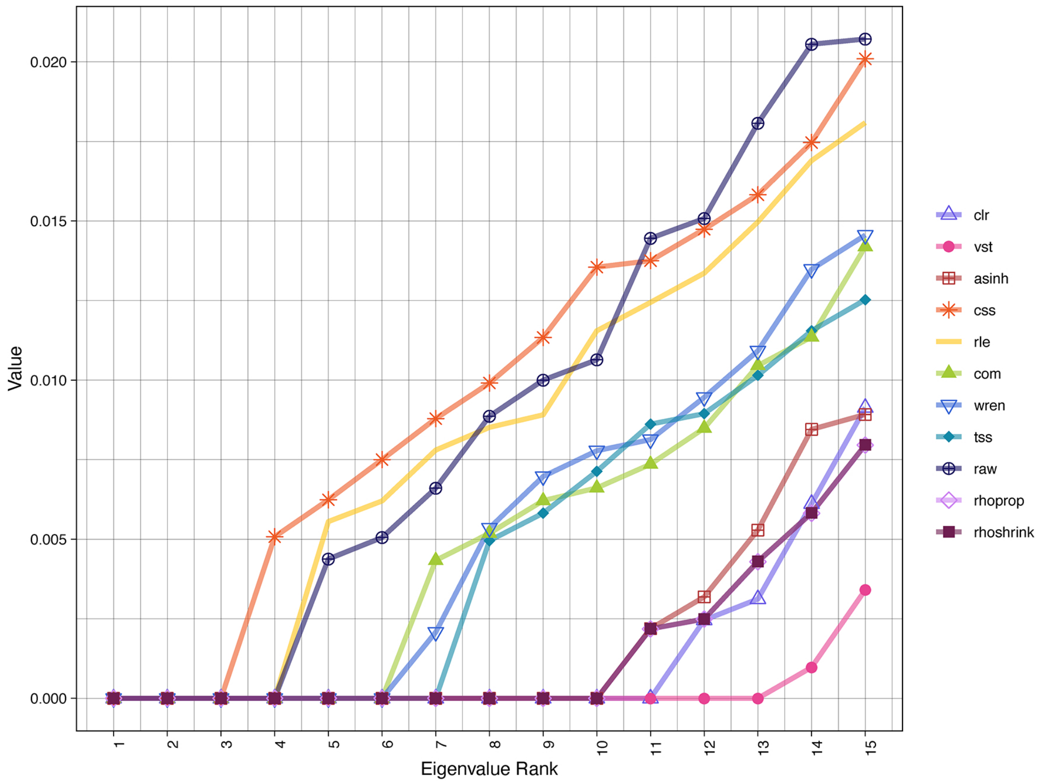

Figure S6: Numbers of clusters as selected by the number of connected components. Vertical lines represent the first non-zero eigenvalue which is selected as the number of clusters.

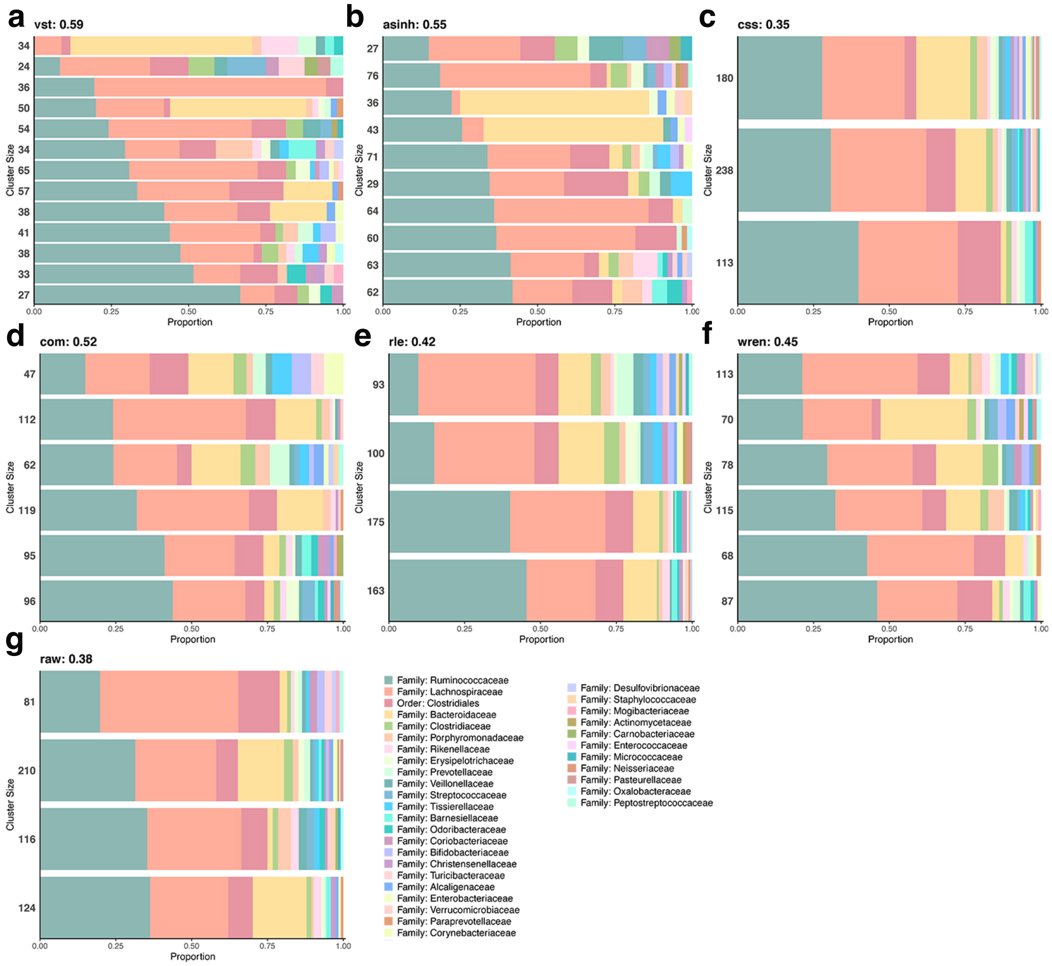

Figure S7: Horizontal stacked bar plot of OTU groups resulting from spectral clustering. The stacked bar plots represent the composition of OTUs in each cluster at the Family level. Clusters are vertically ordered from highest percentage of the most abundant Family: *Ruminococcaceae*. Horizontally the order represents the highest percentage of Family in each cluster. Numbers on the left axis represent the number of OTUs in each cluster. Values stated next to method name represent cluster purity.

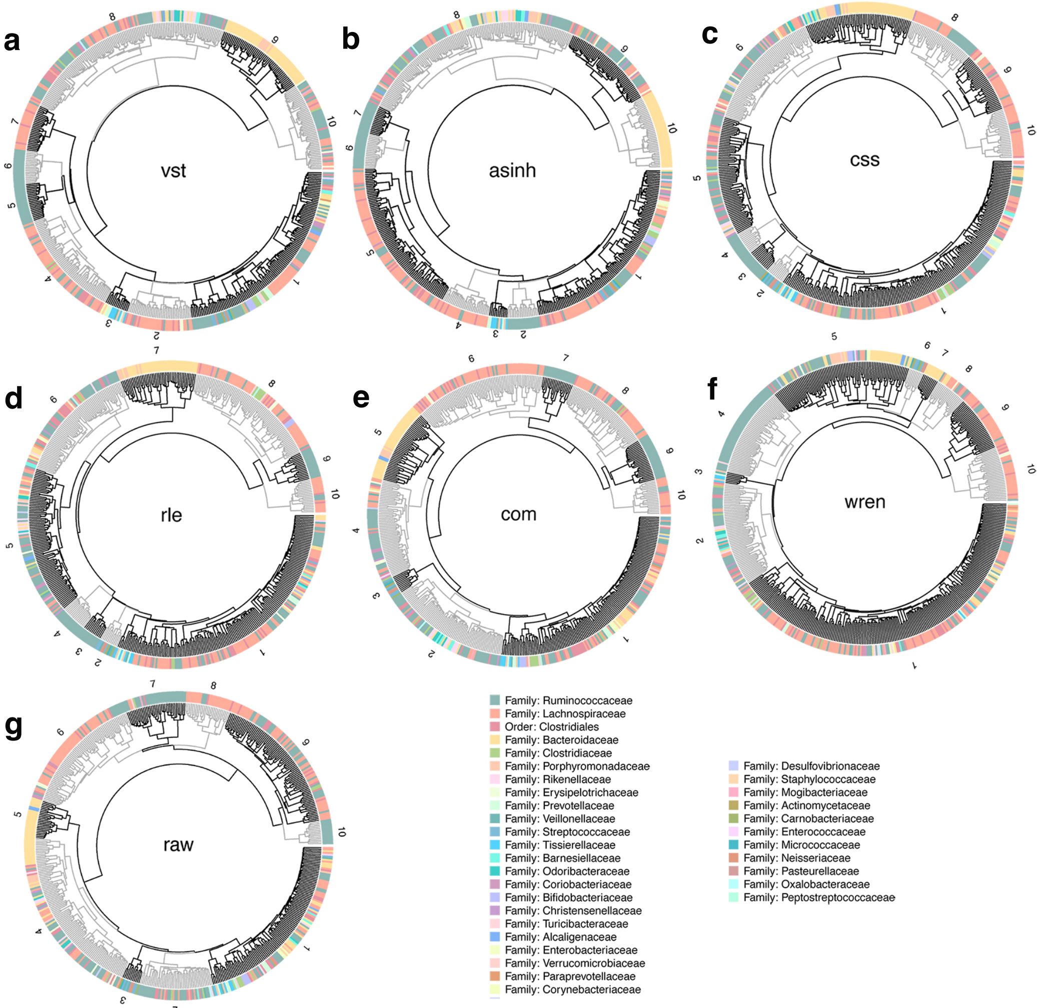

Figure S8: Circular dendrograms showing hierarchical clustering patterns amongst OTUs. Each point surrounding the circular dendrogram represents one of the 531 OTUs in our data set. The color represents Family annotation. Each dendrogram (a-g) has been cut hierarchically into 10 trees (representing the 10 Orders to which these taxonomic Family map). The grey and black shading is used to highlight different clusters which are numbered.

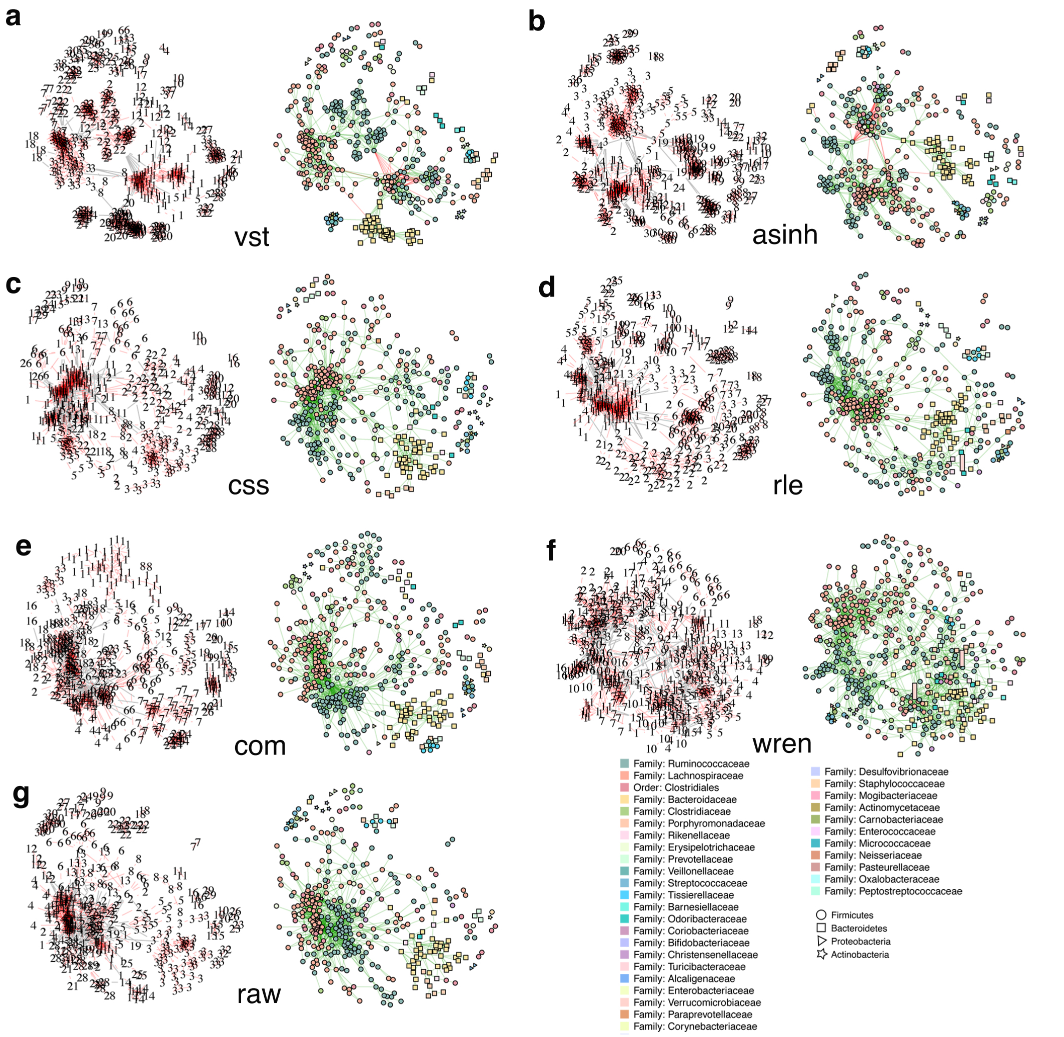

Figure S9: Relevance network visualization displaying modularity. For networks on the left of each panel every node represents an OTU labelled with module annotation as predicted by the Fast-Greedy modularity algorithm. The networks on the right represent the corresponding phylogenetic annotation of the OTU at the family level. Values stated next to method name represent the number of modules in the network. Layout using the force-directed Fruchterman-Reingold algorithm was conserved for both networks in each panel for comparison

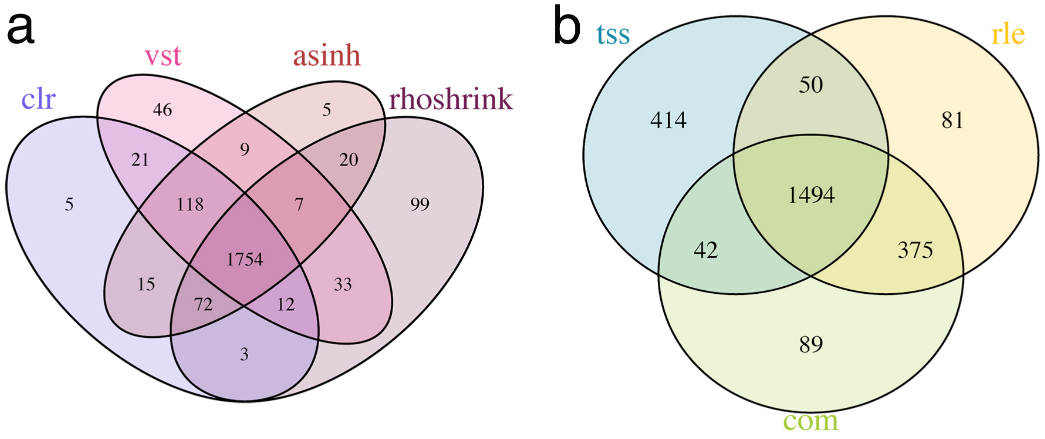

Figure S10: Edges in common between relevance networks. Venn Diagram of edges in common from the top 2000 edges between a) Log-based normalization methods clr, vst, asinh and rhoshrink b) rle, com, and tss.

2 Supplementary Methods

2.1 Shrinkage estimation

$$\widehat{\lambda}_1^* = \min\left(1, \frac{\sum\limits_{i \neq j} \widehat{Var}\left(\hat{r}_{ij}\right)}{\sum\limits_{i \neq j} \left(\hat{r}_{ij}^2\right)}\right)$$

$$\widehat{\lambda}_2^* = \min\left(1, \frac{\sum\limits_{i=1}^{n} \widehat{Var}\left(\hat{s}_{ii}\right)}{\sum\limits_{i=1}^{n} \left(\hat{s}_{ii} - v\right)^2}\right)$$

The detailed computation of unbiased estimation of variance ($\widehat{Var}$) for $\widehat{\lambda}_1^*$ and $\widehat{\lambda}_2^*$ can be found in Schäfer and Strimmer (27, 57).

2.2 Kolmogorov-Smirnov D-statistic

The Kolmogorov-Smirnov (KS) test is a one-sample test that uses the empirical distribution function to compare one-dimensional distributions. We use the D-statistic associated with the KS test to assess whether individual OTU count distributions, after applying a specific transformation, are closer to a normal distribution with parameters specified by the mean and standard deviation of the transformed input. The D-statistic (D for distance) measures the maximal distance between the cumulative density function of the input distribution and the cumulative density function of a normal distribution with the same first and second moment. Here, we use the D-statistic to illustrate the difference between each OTU distribution and a moment-matched normal distribution. A larger D-statistic value thus indicates larger deviation from normality. For each transformation, we report the average D-statistic value across all OTUs in Figure 9.
